# RCS Estimation of Singly Curved Dielectric Shell Structure with PMCHWT Method and Experimental Verification

**DOI:** 10.3390/s22030734

**Published:** 2022-01-19

**Authors:** Hyeong-Rae Im, Woobin Kim, Yeong-Hoon Noh, Ic-Pyo Hong, Jong-Gwan Yook

**Affiliations:** 1 Department of Electrical and Electronic Engineering, Yonsei University, Seoul 03722, Korea; ihr3021@yonsei.ac.kr (H.-R.I.); woobink0203@yonsei.ac.kr (W.K.); yh.noh@yonsei.ac.kr (Y.-H.N.); 2 Department of Smart Information Technology Engineering, Kongju National University, Cheonan 31080, Korea; iphong@kongju.ac.kr

**Keywords:** method of moment (MoM), Poggio–Miller–Chang–Harrington–Wu–Tsai (PMCHWT), singly curved dielectric, radar cross section (RCS), monostatic RCS measurement

## Abstract

In this paper, a numerical algorithm for the electromagnetic scattering analysis of singly curved dielectric structures, which can be applied to a canopy of fighter aircraft, is presented with experimental verification. At first, the Poggio–Miller–Chang–Harrington–Wu–Tsai (PMCHWT) method is used as a MoM-based solution for the electromagnetic scattering of a dielectric material. Its formulation was generated with the EFIE formulation in a multi-region condition. The PMCHWT algorithm is implemented with C++ code, and the accuracy is verified by calculating the bistatic RCS of some canonical structures with conductive or dielectric materials. RCS measurement under quasi-anechoic condition is presented with its procedure and calibration method. The monostatic RCS results of a specially modeled singly curved dielectric structures are obtained analytically with the PMCHWT, as well as experimentally, revealing excellent agreement.

## 1. Introduction

When it comes to modern aerospace, as well as naval systems for defense applications, the low-observability against radar is one of the most important requirements to improve the survivability of the system. The radar cross-section (RCS) of a system can be estimated from the measurements in a near-field or far-field condition [1]. On the other hand, the computational electromagnetic technologies have been widely utilized to make the process of RCS estimation more manageable, as far as possible [2,3,4,5,6]. In many practical situations, measurement is not possible, especially for electrically large objects.

In the numerical estimation of RCS, the method of moment (MoM) is one of the rigorous numerical solutions [7]. In general, the MoM with surface integral equation (SIE) is a better approach than with the volume integral equation (VIE) when the object under estimation is electrically large [8]. In recent studies, many efficient MoM-based algorithms have been proposed as a solution for large-scale problems. In many commercial and industrial approaches, the multi-level-based MoM solutions have proved their effectiveness at handling large-scale problems [3,9,10]. Even further, the fast multi-pole method (FMM) [11] and characteristic basis function method(CBFM) [12,13] can be combined with the multi-level algorithms.

To analyze the electromagnetic scattering property of dielectric or composite objects, many combinations of SIE and VIE approaches have been proposed and applied to practical cases. Among them, the Poggio–Miller–Chang–Harrington–Wu–Tsai (PMCHWT) formulation has been popularly used, because of the accuracy and stability of the results at arbitrarily shaped objects [8]. In addition, the Carderon preconditioner has been used with it for the acceleration of the iterative solver [14,15].

For the stealth characteristic of the fighter aircraft, several technologies have been researched and applied to it. The radar absorbing material (RAM) and radar absorbing structure (RAS) are two popular techniques of them [16]. In particular, the canopy of the aircraft, a protection structure for pilots, is also the object of RCS reduction. For a typical aircraft, the canopy is composed of a dielectric material with high stiffness and strength properties. In terms of electromagnetic field, it has a low level of RCS. However, it also has high electromagnetic transparency, so that the RCS of the inner cockpit has large scattering as a corner reflector. An acrylic-based material with a thin multilayered surface composed of a transparent conductive oxide (TCO) has been researched for its microwave absorption properties [17,18]. To reduce the RCS of an aircraft, a proper method for the RCS reduction of a singly curved dielectric object is required.

In this paper, the RCS of a singly curved dielectric is estimated and discussed by using both a numerical method and a measurement. In Section 2, PMCHWT, which is a MoM-based numerical algorithm for analyzing the scattering property of dielectric objects, is formulated for a dielectric scatterer. In Section 3, an RCS measurement setup at the quasi-anechoic circumstance is presented, and RCS calibration is verified. In Section 4, the PMCHWT algorithm is verified by some examples with canonical scatterers. In addition, a singly curved dielectric object with a specific curvature is numerically calculated by the PMCHWT in-house algorithm. The electromagnetic scattering level of the same object as a device under test is measured and its RCS is yielded. The numerical and experimental RCS data are compared and discussed.

## 2. PMCHWT Formulation

**Figure 1:**
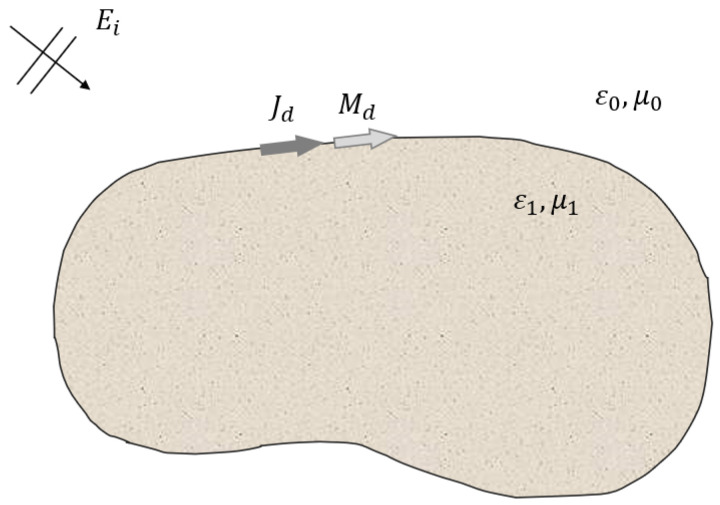
Equivalent electric and magnetic currents on the boundary of the dielectric object.

To obtain scattering properties of a dielectric structure with MoM, the PMCHWT algorithm is formulated on the surface boundary mesh between two piece-wise homogeneous regions [11]. Figure 1 shows a dielectric object under plane wave excitation. For a dielectric object with a surface Γ, the equivalent electric and magnetic currents, J and M, are modeled as unknowns of the SIE. For the incident electric and magentic field, $E^{p}$ and $H^{p}$, each of the equations can be written as follows.

(1) $$\frac{\eta }{jk}\gamma_{t}L\left(J\right)-\gamma_{t}K\left(M\right)-\frac{1}{2}\gamma_{r}M=\gamma_{t}E^{p}$$

(2) $$\gamma_{t}K\left(J\right)-\frac{1}{2}\gamma_{r}J+\frac{1}{jk\eta }\gamma_{t}L\left(M\right)=\gamma_{t}H^{p}$$

where *k* and η are the wave constant and wave impedance of the region of the problem. The integro-differential operators $L_{\Gamma }$ and $K_{\Gamma }$ are defined as

(3) $$L_{\Gamma }\left[F\left(r\right)\right]=\left(\nabla \nabla \cdot +k^{2}\right)\int_{\Gamma }G\left(r,r^{\prime }\right)F_{\Gamma }\left(r^{\prime }\right)d\Gamma^{\prime }$$

(4) $$K_{\Gamma }\left[F\left(r\right)\right]=\nabla \times \int_{\Gamma }G\left(r,r^{\prime }\right)F_{\Gamma }\left(r^{\prime }\right)d\Gamma^{\prime }$$

where $G(r,r^{\prime })$ is Green’s function for the region of problem. In addition, $\gamma_{t}$ and $\gamma_{r}$ are defined as

(5) $$\gamma_{t}\left(F\right)=-n\times n\times F$$

(6) $$\gamma_{r}\left(F\right)=n\times F$$

The Formulation (1) and (2) are called the electric field integral equation (EFIE) and magnetic field integral equation (MFIE), respectively. If a homogeneous dielectric material is the object of interest for a scattering problem, then there are two different regions; that is, the interior and exterior region of the object. The SIE-MoM formulation is based on the homogeneous region. It produces two different EFIEs and MFIEs which have to be satisfied at each region as summarized below.

(7) $$\frac{\eta_{1}}{jk_{1}}\gamma_{t}L_{1}\left(J_{1}\right)-\gamma_{t}K_{1}\left(M_{1}\right)-\frac{1}{2}\gamma_{r}M_{1}=\gamma_{t}E^{p}$$

(8) $$\frac{\eta_{2}}{jk_{2}}\gamma_{t}L_{2}\left(J_{2}\right)-\gamma_{t}K_{2}\left(M_{2}\right)-\frac{1}{2}\gamma_{r}M_{2}=0$$

(9) $$\gamma_{t}K_{1}\left(J_{1}\right)-\frac{1}{2}\gamma_{r}J_{1}+\frac{1}{jk_{1}\eta_{1}}\gamma_{t}L_{1}\left(M_{1}\right)=\gamma_{t}H^{p}$$

(10) $$\gamma_{t}K_{2}\left(J_{2}\right)-\frac{1}{2}\gamma_{r}J_{2}+\frac{1}{jk_{2}\eta_{2}}\gamma_{t}L_{2}\left(M_{2}\right)=0$$

In the Equations (1) and (2), the direction of surface currents are dependent to outer normal vector **n** so that $J_{1}=-J_{2}$ and $M_{1}=-M_{2}$. In addition, the signs of the $\gamma_{t}$ and $\gamma_{r}$ operator have to be considered. In (8) and (10), the right-hand side is zero, because the incident fields are assumed to exist in the free space only. As the results of a combination of the equations, there are two independent integral equations to be solved.

(11) $$[\frac{\eta_{1}}{jk_{1}}\gamma_{t}L_{1}+\frac{\eta_{2}}{jk_{2}}\gamma_{t}L_{2}]\left(J_{1}\right)-\left[\gamma_{t}K_{1}+\gamma_{t}K_{2}\right]\left(M_{1}\right)=\gamma_{t}E^{p}$$

(12) $$\left[\gamma_{t}K_{1}+\gamma_{t}K_{2}\right]\left(J_{1}\right)+[\frac{1}{jk_{1}\eta_{1}}\gamma_{t}L_{1}+\frac{1}{jk_{2}\eta_{2}}\gamma_{t}L_{2}]\left(M_{1}\right)=\gamma_{t}H^{p}$$

Finally, there are two equations with two common unknowns, and they can be solved by matrix inversion or the iterative method in the MoM concept. The scattering level of the electromagnetic field by the dielectric object can be obtained by post-processing from the resulting electric and magnetic currents.

## 3. Monostatic RCS Measurement

The monostatic as well as bistatic RCS of an object can be measured with various facilities based on near- and far-field circumstances. As an important preparation, the measurement environment should minimize the uncertainty and error in the measured data. In general, most RCS measurement facilities are implemented under anechoic chamber conditions to exclude the effects of clutters and unwanted scatterers. In this paper, a quasi-anechoic measurement environment is utilized and an approximated comparison has been performed between the numerical and experimental data sets.

**Figure 2:**
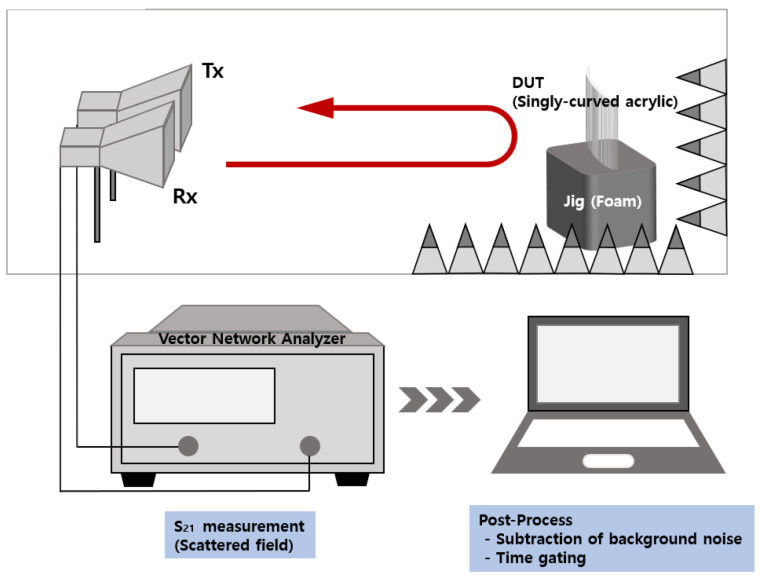
Setup of monostatic RCS measurement with Tx/Rx condition.

Figure 2 illustrates a setup of an RCS measurement performed in this paper. Two antennas at the target frequency band are used to obtain the transferred signals from one to the other. This signal contains several scattering components from the objects in the beam region of the antenna. To measure the scattered fields of the object under test only, the appropriate placement of absorbers are necessary. The OUT is placed on top of the styrofoam block and the laser leveling tool is used for accurate positioning. For minimizing the positioning error, the OUT is placed along the center of the main beam of the Tx and Rx antennas. The transmitting and receiving antennas are connected with a vector network analyzer, and scattering parameters are measured.

The monostatic RCS of a scatterer can be estimated from the measured data after applying several post-processings. First of all, RCS calibration data are required to compensate the environmental effects of the measurement system. A flat square metallic plate with a square or spherical shape is a standard calibration target. The verification of the measurement system is performed by comparing the scattered field from an analytic or numerical calculation and the measurement for a well-known target. In addition, to cancel out the signals other than the OUT, the scattering signal in a free space is needed. This cancellation requires quite a low level of RCS to be measured with higher accuracy. Another step is the time-gating process, which means a filtering of a specific range of the time domain data. It is to extract the component of scattering field data caused by the target object. Therefore, the span of the filter should be decided as a minimum value which contains the entire range of the target with a distance.

**Figure 3:**
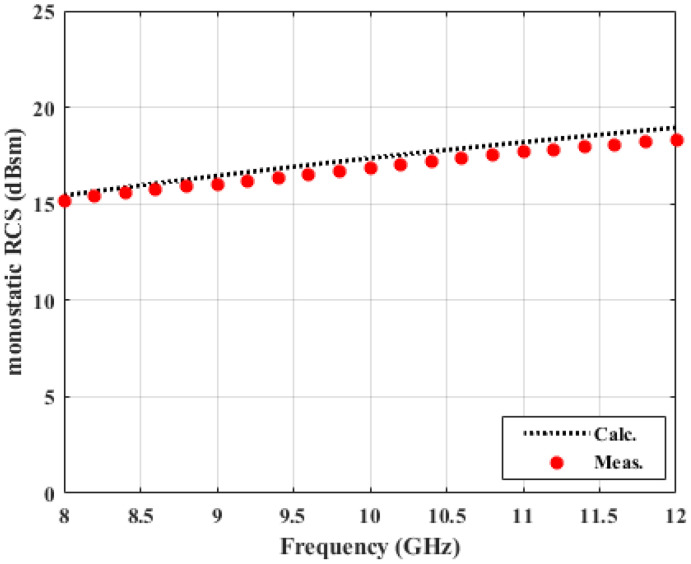
Monostatic RCS calibration (copper plate, 20 cm × 20 cm).

Figure 3 shows a monostatic RCS of a square-shaped copper plate with side length of 20 cm. It is clear that the RCS calibration process is well done, based on the excellent agreement between the calculated data and the measured (after calibration) data. For this calibration process, a 15 cm square-shaped one is used as a calibration target.

## 4. Results and Discussion

### 4.1. MoM Solution of Canonical Structure

**Figure 4:**
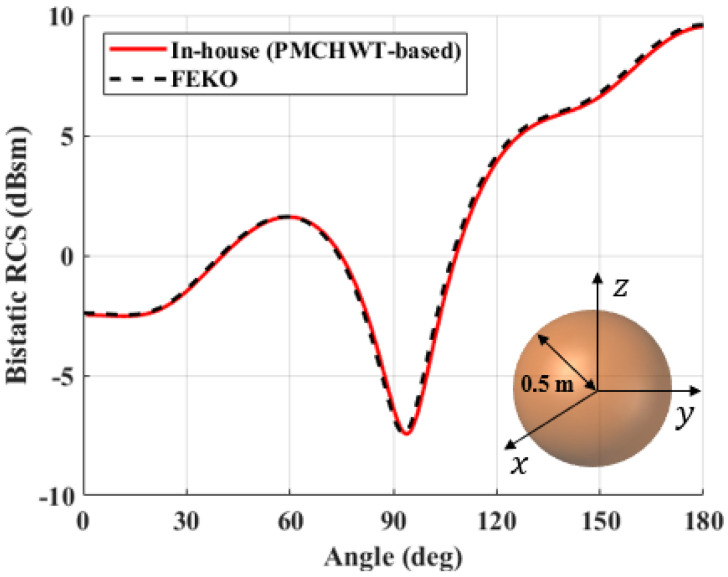
Bistatic RCS of a PEC sphere with radius of 0.5 m.

**Figure 5:**
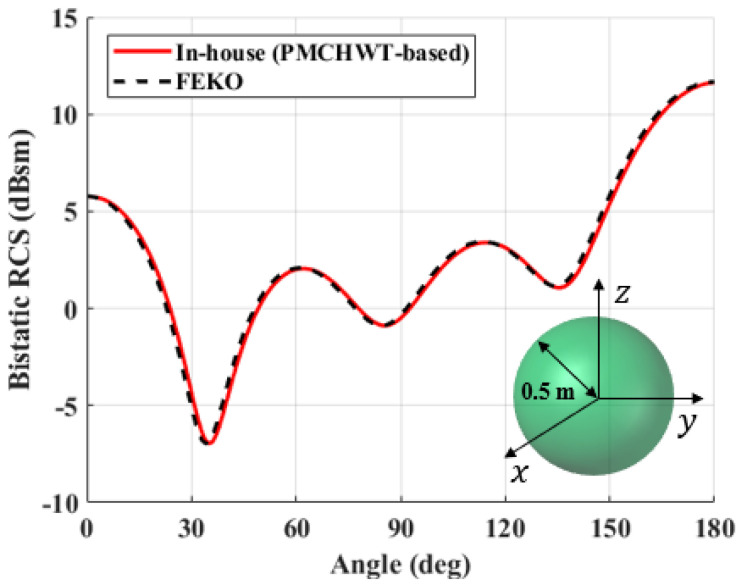
Bistatic RCS of a dielectric sphere with radius of 0.5 m.

**Figure 6:**
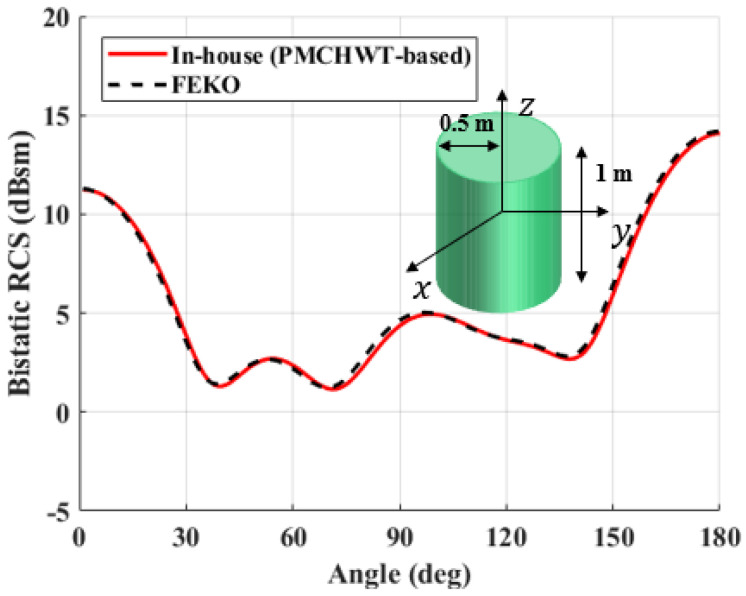
Bistatic RCS of a dielectric cylinder with radius of 0.5 m and height of 1 m.

To verify the accuracy of the PMCHWT-MoM solution developed in this study, the solutions are compared with commercial software (FEKO) for three different problems. Three different canonical structures; perfect electric conductoring sphere, dielectric sphere, and a dielectric cylinder structure; are used for the verification. The dielectric constant is 4 without loss. The x-polarized electric field is excited at 300 MHz, and the bistatic RCS values along the elevation angle (theta) of the spherical coordinate are evaluated. The RCS of the PEC and dielectric sphere with a radius of 0.5 m are shown in Figure 4 and Figure 5. For the PEC structure, the EFIE formulation is used. Meanwhile, Figure 6 shows the RCS of a dielectric cylindrical structure. It is clear that the PMCHWT-based MoM solution yields excellent agreement with commerial software data.

### 4.2. MoM Solution of a Singly Curved Dielectric Shell

**Figure 7:**
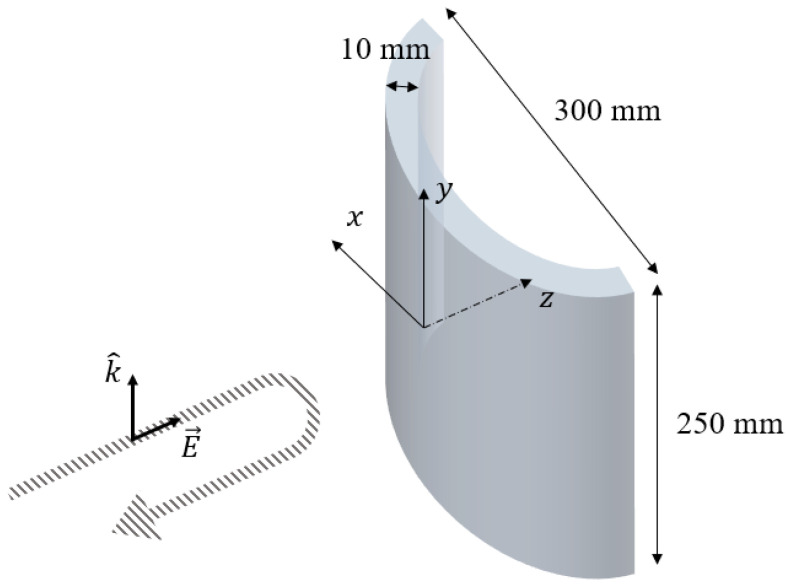
A singly curved dielectric for RCS Estimation.

**Figure 8:**
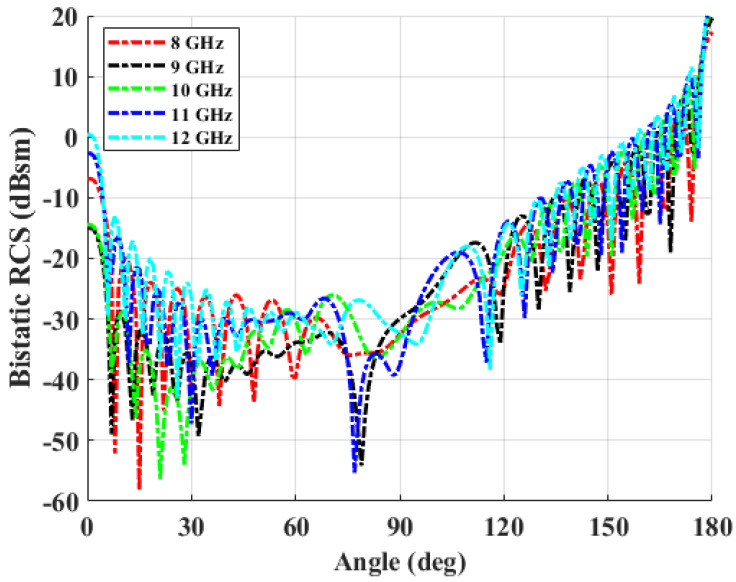
Bistatic RCS of singly curved dielectric sample.

In order to obtain the scattering characteristics of a curved dielectric, a singly curved dielectric shell is considered. The shape of the sample is shown in Figure 7. The length of the arc and the side of OUT are 300 mm and 250 mm, respectively, as illustrated in the figure. The radius of the curvature is 369.9 mm. The permittivity and loss constants of the dielectric shell are 2.45 and 0.0052, and the thickness is 10 mm. In the work, RCS is calculated in the frequency of 8 to 12 GHz. There are 17,778 edges in the mesh, and there are twice the number of unknowns of the electric and magnetic currents, which are the coefficients of the basis functions. The bistatic RCS characteristics of the dielectric shell are shown in Figure 8. The polarization of incident field is set as the unit normal vector to the ground, which is $\hat{y}$, and the received signals of co-polarization as well as cross-polarization are calculated. It is interesting to note that the forward scattering level is about 20 dB higher than the back scattering level, due to the use of low-loss, singly curved acrylic material.

### 4.3. Monostatic RCS Measurement of a Singly Curved Dielectric Shell

**Figure 9:**
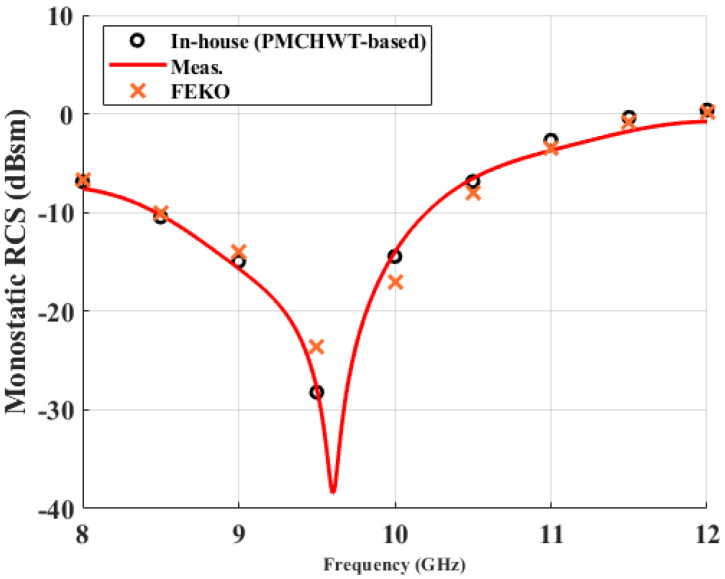
Monostatic RCS of singly curved dielectric shell sample.

The monostatic RCS of the singly curved dielectric is measured in a non-anechoic condition, as mentioned in the previous section. The transmitted signal of an aluminum plate with a side length of 25 cm is used as a calibration target. Figure 9 shows the measured and calculated monostatic RCS. It can be shown that there is a null point of RCS at close to 9.6 GHz. The frequency of the null point is determined by the permittivity and thickness of the OUT. It is noteworthy that the measured as well as the calculated RCS values agree very well, even in the low-level regime. In view of electromagentic scattering, a singly curved structure does not support a strong creeping wave as in the sphere or cylinder cases. Most of the scattered field is due to reflection, refraction, and transmission.

## 5. Conclusions

The electromagnetic scattering from a singly curved dielectric has been predicted with the PMCHWT-based method of moment, as well as from measurement in a quasi-anechoic chamber environment. For analyzing the RCS characteristics of an object, the PMCHWT algorithm has been developed as a MoM-based solution. The PMCHWT formulation was constructed by combining the EFIEs and MFIEs for all regions generated in space by the target. The accuracies of the EFIE and the PMCHWT algorithm used in this paper have been verified by comparing the RCS solutions of some commericial EM software for sphere and cylinder forms with doubly and singly curved shapes. A special case of a singly curved dielectric shell is implemented as an OUT of monostatic RCS measurement. A calibration method with a scattering of a flat metal plate has been used. In addition, the subtraction of scattering fields from clutters and time-gating at a proper distance have been applied as a post-processing of measured data. The comparison of the PMCHWT solution and the measured data shows the accuracy of both the measurement as well as the computational tool. As further research, some MoM-based methods for large-scale analysis can be combined with EFIE-PMCHWT for an accurate and more accelerated estimation of electromagnetic scatterings. In addition, more practical studies are also planned to be performed, such as the RCS analysis of some curved dielectric structure with lossy layer coating, which can be applied to aircraft.

## Data Availability

Not applicable.
